# Evaluation of Qualitative Changes in Simulated Periodontal Ligament and Alveolar Bone Using a Noncontact Electromagnetic Vibration Device with a Laser Displacement Sensor

**DOI:** 10.1155/2016/9636513

**Published:** 2016-05-04

**Authors:** Hiroshi Kobayashi, Makoto Hayashi, Masaru Yamaoka, Takuya Yasukawa, Haruna Ibi, Bunnai Ogiso

**Affiliations:** ^1^Department of Endodontics, Nihon University School of Dentistry, 1-8-13 Kanda-Surugadai, Chiyoda-ku, Tokyo 101-8310, Japan; ^2^Division of Advanced Dental Treatment, Dental Research Center, Nihon University School of Dentistry, 1-8-13 Kanda-Surugadai, Chiyoda-ku, Tokyo 101-8310, Japan; ^3^Department of Physics, Nihon University School of Dentistry, 1-8-13 Kanda-Surugadai, Chiyoda-ku, Tokyo 101-8310, Japan; ^4^Division of Applied Oral Sciences, Nihon University Graduate School of Dentistry, 1-8-13 Kanda-Surugadai, Chiyoda-ku, Tokyo 101-8310, Japan

## Abstract

Evaluating periodontal tissue condition is an important diagnostic parameter in periodontal disease. Noncontact electromagnetic vibration device (NEVD) was previously developed to monitor this condition using mechanical parameters. However, this system requires accelerometer on the target tooth. This study assessed application of laser displacement sensor (LDS) to NEVD without accelerometer using experimental tooth models. Tooth models consisted of cylindrical rod, a tissue conditioner, and polyurethane or polyurethane foam to simulate tooth, periodontal ligament, and alveolar bone, respectively. Tissue conditioner was prepared by mixing various volumes of liquid with powder. Mechanical parameters (resonant frequency, elastic modulus, and coefficient of viscosity) were assessed using NEVD with the following methods: Group A, measurement with accelerometer; Group B, measurement with LDS in the presence of accelerometer; and Group C, measurement with LDS in the absence of accelerometer. Mechanical parameters significantly decreased with increasing liquid volume. Significant differences were also observed between the polyurethane and polyurethane foam models. Meanwhile, no statistically significant differences were observed between Groups A and B; however, most mechanical parameters in Group C were significantly larger and more distinguishable than those of Groups A and B. LDS could measure mechanical parameters more accurately and clearly distinguished the different periodontal ligament and alveolar bone conditions.

## 1. Introduction

Periodontal disease such as gingivitis and periodontitis has damaging effects on the periodontal tissue due to bacterial infection. Especially in periodontitis, loss of the periodontal tissue including periodontal ligament and alveolar bone that surround and support the teeth is involved [1, 2]. Tooth mobility basically increases in proportion to the progression of periodontitis; therefore, the measurement and monitoring of tooth mobility are an important diagnostic tool for evaluating tooth and periodontal tissue condition [3, 4].

Traditionally, Miller's classification [5] that assesses manual movement with two rigid instruments has been used for this purpose, because of its simplicity and practicality in the clinical situation. However, this method is a subjective technique and not always precise, depending upon the dentist's tactile sense and experience [6, 7].

Periodontal tissue, particularly the periodontal ligament, has elastic and viscous properties because periodontal ligament has a complex structure which consists of collagen fiber, nerves, blood vessels, and fluid [3, 8, 9]. These properties lead to difficulties in the evaluation of the periodontal tissue condition including tooth mobility, since the elastic and viscous properties are responsible for the nonlinear and time-dependent behaviour of the periodontal ligament [3].

A noncontact electromagnetic vibration device was developed to objectively and accurately assess the overall periodontal tissue condition [10–12]. This device analyzes both tooth mobility and the periodontal tissue condition using mechanical parameters, that is, resonant frequency, elastic modulus, and coefficient of viscosity, by measuring the vibration of the tooth using an electromagnetic force. As previously described, the NEVD can accurately assess the bottom thickness and qualitative changes of a simulated periodontal ligament and alveolar bone in an experimental tooth model [10, 11]. Additionally, this device could not only monitor the periodontal tissue condition but also implant stability using the same mechanical parameters [12]. Although these results indicate the application of NEVD to the evaluation of periodontal tissue conditions, this system requires the attachment of an accelerometer to the target tooth to detect the vibration. Then, the accelerometer must be connected to the fast Fourier transformation (FFT) analyzer by cord. This cord may influence the precise detection of mechanical parameters.

A displacement sensor can measure the distance to the target object, and there are two types of sensors: contact type and noncontact type. One of the noncontact types is a laser displacement sensor (LDS), which can measure the distance as well as the acceleration of the target object without mechanical contact (cordless) [13–15]. A recent study [16] revealed that the application of the LDS, instead of the accelerometer, to NEVD can detect the different degrees of simulated bone destruction and qualities using the* in vitro* experimental tooth models.

It is unknown whether the use of LDS with NEVD can detect the different condition of simulated periodontal ligament and alveolar bone. In this study, it was hypothesized that NEVD with LDS could detect qualitative changes in a simulated periodontal ligament and alveolar bone using mechanical parameters.

## 2. Materials and Methods

### 2.1. *In Vitro* Experimental Tooth Model

**Figure 1:**
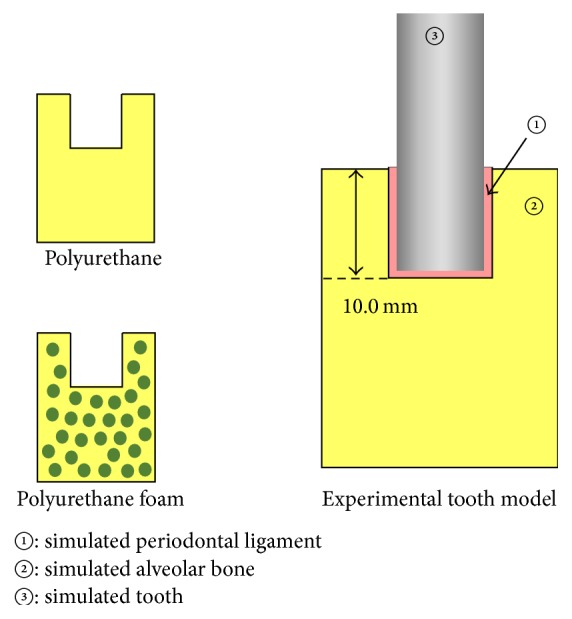
Design of the experimental tooth models.

A cylindrical rod made of polyacetal (diameter × length: 6.0 × 25.0 mm, mass: 1.59 g), a tissue conditioner designed for functional impression (Shofu Tissue Conditioner II; Shofu Inc., Kyoto, Japan), and a block of polyurethane or polyurethane foam (Nissin Dental Products Inc., Kyoto, Japan) were used to simulate a tooth, a periodontal ligament, and an alveolar bone, respectively. Experimental tooth models were developed using procedures modified from a previous method [10]. Briefly, simulated teeth (polyacetal) were submerged 10.0 mm into simulated bones (polyurethane or polyurethane foam) containing simulated periodontal ligaments (tissue conditioner) of 0.5 mm in thickness (Figure 1). All of the experimental models were set up in thermohygrostat room (temperature: 23 ± 1°C; relative humidity: 50 ± 5%). The models were maintained under these conditions for 1 h prior to each measurement.

### 2.2. Experimental Conditions of Simulated Periodontal Ligament and Alveolar Bone

Simulated periodontal ligament was prepared using various liquid volumes of the tissue conditioner, according to previously described methods [10]. In brief, the standard liquid volume for clinical use of the soft lining material according to the manufacturer's instructions is 4.0 mL of liquid with 4.8 g of powder. In this study, three different simulated periodontal ligaments were prepared by mixing 3.0, 4.0, and 5.0 mL of liquid with 4.8 g of powder. Simulated alveolar bone was made of polyurethane or polyurethane foam.

### 2.3. NEVD System

The NEVD system was comprised of three components: a vibrator, a detector, and an analyzer as previously described [10–12]. Briefly, the vibrator consisted of a ferrite disk magnet (maximum magnetic flux: 130 mT, mass: 0.19 g, and diameter: 5.2 mm) (PIP Co., Ltd., Osaka, Japan), an electromagnetic vibration device, and a sensor amplifier (Toshiba TA7252AP Audio Amplifier Kit; Akizuki Denshi Tsusho Co., Ltd., Tokyo, Japan). The ferrite disk magnet was attached to the lateral surface at the top of the simulated tooth by an adhesive (cyanoacrylate; Toagousei Co., Ltd., Tokyo, Japan). The ferrite disk magnet received an electrical force generated by the alternating magnetic field produced by the electromagnetic vibration device. The electromagnetic vibration device consisted of a ferrite rod wrapped with enamel wire (diameter: 5.0 mm) 720 times to form a coil.

### 2.4. Detection Methods

Three detection methods were used to assess the vibration of simulated teeth in NEVD system: Group A, a conventional measurement of tooth vibration using an accelerometer (mass: 0.40 g) (NP-3211; Ono Sokki Co., Ltd., Tokyo, Japan) attached to the simulated tooth; Group B, a measurement of tooth vibration using LDS in the presence of an accelerometer attached to the simulated tooth; Group C, a measurement of tooth vibration using LDS without an accelerometer.

**Figure 2:**
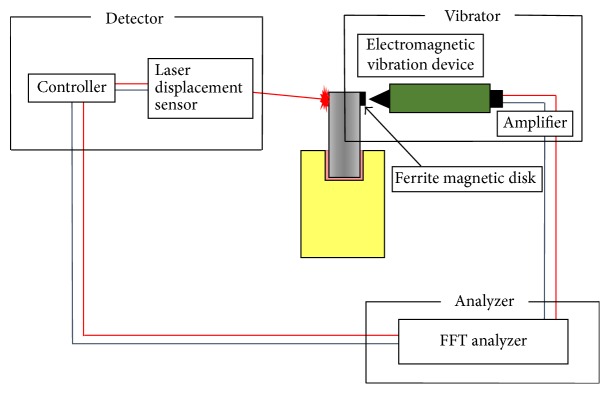
Schematic representation of the experimental design for the NEVD system with LDS.

The schematic representation of the experimental design for Group C is shown in Figure 2. The LDS was placed 50 mm away from the simulated tooth. For optimal laser reflection, aluminum foil (5.0 × 5.0 mm) was attached to the lateral surface at the top of the simulated tooth. Vibration of the simulated tooth was detected by the LDS (repeatability: 0.025 *μ*m) (LK-H055; Keyence Corp., Osaka, Japan) equipped with a red laser diode (wavelength: 655 nm; power: 4.8 mW). The output signal from the LDS was input to the FFT analyzer via the controller (LK-G5000; Keyence Corp.) that was connected to the LDS. The frequency-response characteristics for the experimental tooth model (i.e., the ratio between the output of a sweep generator and the input of the LDS) were calculated by the FFT analyzer. With a frequency resolution of 12.5 Hz and 80 ms capture time, measurements were made over a frequency range of 5 kHz.

**Figure 3:**
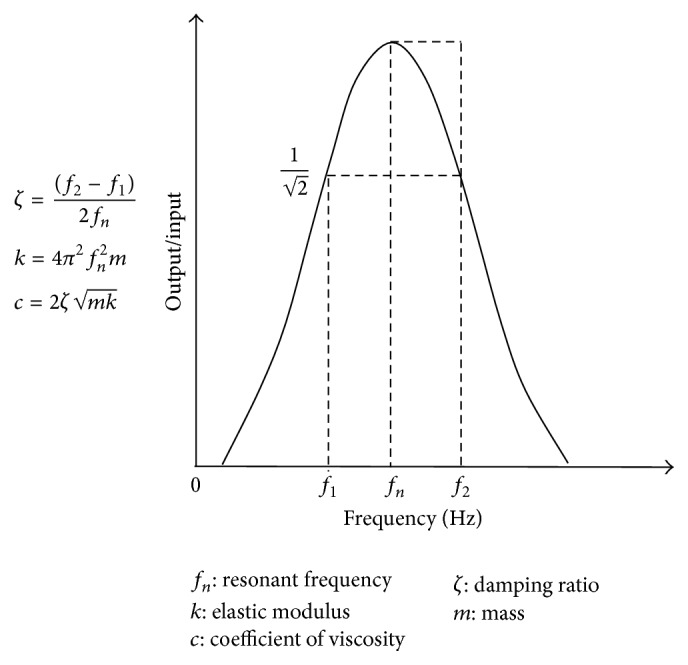
Frequency-response characteristics and formulae for calculation of the mechanical parameters. *f*_1_ and *f*_2_ are the frequencies at $1/\sqrt{2}$ times the maximum amplitude of the resonant frequency (*f*_*n*_).

Mechanical parameters were calculated as previously described (Figure 3) [10]. Five experimental tooth models were analyzed for each condition (*n* = 5). Data are expressed as the median plus the maximum and minimum value for mechanical parameters.

### 2.5. Statistical Analyses

Differences between mechanical parameters among different periodontal ligament conditions or different detection methods were performed using the Kruskal-Wallis and Steel-Dwass tests. In addition, the differences between the two types of simulated bone quality were compared using the Mann-Whitney *U* test. Values of *P* < 0.05 were considered significant.

## 3. Results

### 3.1. Resonant Frequency

**Figure 4:**
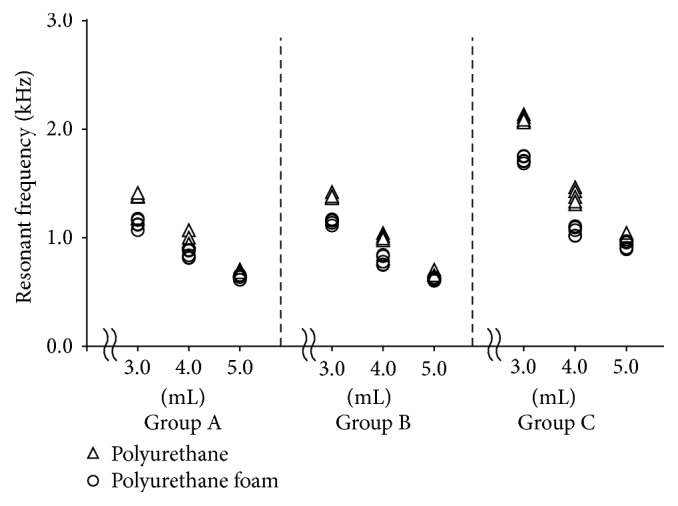
Resonant frequency at different liquid volumes and simulated bone qualities using experimental tooth models. Triangles indicate the urethane model while circles indicate the urethane foam model.

The resonant frequency for each group is shown in Table 1 and Figure 4. The resonant frequency decreased curvilinearly with increasing liquid volume for the simulated periodontal ligament in both the polyurethane and polyurethane foam models. Significant differences were also observed among the different liquid volumes. In addition, the resonant frequency of the polyurethane model was significantly larger than that of the polyurethane foam model for all simulated periodontal ligament conditions.

### 3.2. Elastic Modulus

**Figure 5 fig5:**
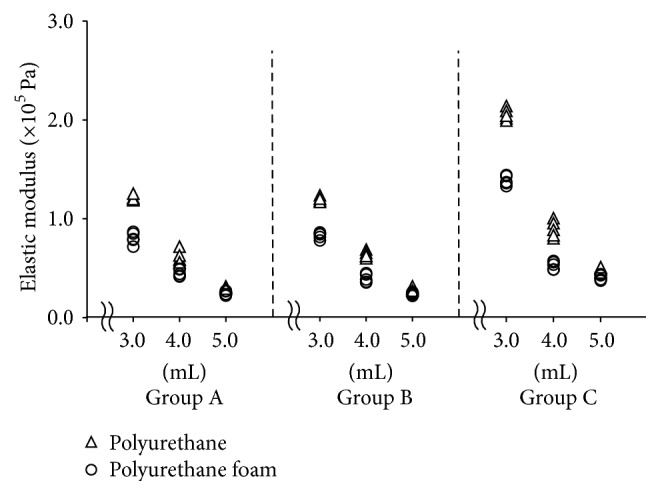
Elastic modulus at different liquid volumes and simulated bone qualities using experimental tooth models. Triangles indicate the urethane model while circles indicate the urethane foam model.

The elastic modulus for each group is shown in Table 2 and Figure 5. Similar to the resonant frequency, the elastic modulus decreased curvilinearly with increasing liquid volume for the simulated periodontal ligament in both the polyurethane and polyurethane foam models. Statistically significant differences were also found among the different liquid volumes, and the elastic modulus of polyurethane model was significantly larger than that of polyurethane foam model for all simulated periodontal ligament conditions.

### 3.3. Coefficient of Viscosity

**Figure 6:**
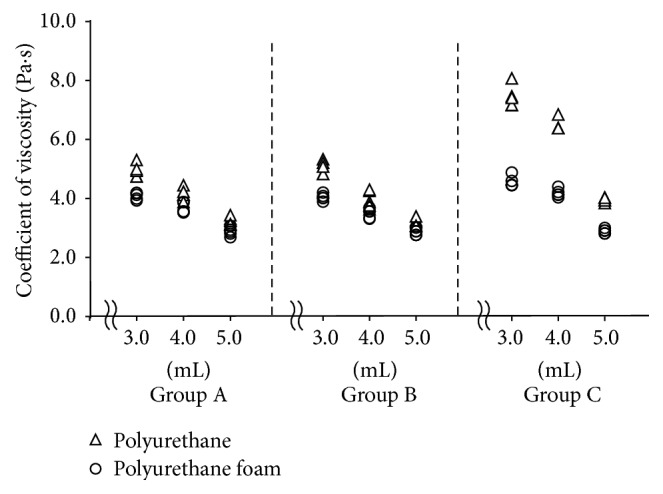
Coefficients of viscosity at different submerged depths and simulated bone qualities using experimental tooth models. Triangles indicate the urethane model while circles indicate the urethane foam model.

The coefficient of viscosity for each group is shown in Table 3 and Figure 6. The coefficient of viscosity decreased linearly with increasing liquid volume for the simulated periodontal ligament in both the polyurethane and polyurethane foam models. Statistically significant differences were also observed among the different liquid volumes, and the coefficient of viscosity for the polyurethane models was significantly larger than that of polyurethane foam model under all simulated periodontal ligament conditions.

### 3.4. Detection Methods

No significant differences were observed for the three mechanical parameters between Groups A and B in all experimental conditions. However, in Group C, the mechanical parameters were significantly larger than those of Groups A and B, except for the elastic modulus in liquid volume of 4.0 mL and the coefficient of viscosity in liquid volume of 5.0 mL for polyurethane foam models (Tables 1 [Table tab2]–3). In addition, the values of the mechanical parameters in Group C were clearly different in various periodontal tissue conditions, so that the values of Group C could be identified very easily as compared with those of Groups A and B (Figures 4 [Fig fig5]–6).

## 4. Discussion

Periodontal disease has an important effect on the periodontal tissue condition and may lead to the loss of teeth. The change of periodontal tissue condition during the progression of periodontal disease influences teeth mobility and is reflected in a change of the mechanical properties of the periodontal tissues [1]. Therefore, the evaluation of the biomechanical characteristics of periodontal tissues may help diagnose periodontal disease [3, 4].

The Periotest is a diagnostic device used for the objective measurement of tooth mobility [5, 17, 18]. The measurement principle of Periotest is the assessment of the time of contact between a percussion rod of the device and the target tooth surface; then, its time is automatically converted into Periotest values from −80 to +50 [5, 17]. The Periotest can be used in daily clinical practice of not only periodontics [19] but also dental implantology [20] and traumatology [6] owing to its simplicity and reproducibility. However, it is difficult to precisely examine the periodontal tissue by Periotest values, because periodontal ligament involves elastic as well as viscous properties [3, 8, 9]. Thus, NEVD was previously developed to assess the overall periodontal tissue condition [10–12].

In this study, the application of LDS to NEVD for the evaluation of simulated periodontal ligament and alveolar bone conditions was examined using the* in vitro* experimental tooth models. Three mechanical parameters (resonant frequency, elastic modulus, and coefficient of viscosity) decreased with increasing liquid volume in both the polyurethane and polyurethane foam models in all detection methods. Kobayashi et al. [10] described the fact that the same mechanical parameters detected the qualitative changes in the simulated periodontal ligament and alveolar bone using NEVD with accelerometer. Their results are consistent with the present study (Group A). In addition, no significant difference between Group A (measurement with accelerometer) and Group B (measurement with LDS in the presence of accelerometer) was observed in this study. Thus, the values in the detection method of LDS have the same quality as those in the detection method of accelerometer, and consequently the reliability of LDS in NEVD was confirmed in the present experimental condition.

Interestingly, values of Group C (measurements with LDS in the absence of accelerometer) were significantly larger than those of Groups A and B for most mechanical parameters. In addition, the values for Group C more clearly distinguished the different periodontal ligament and alveolar bone conditions as compared with Groups A and B (Figures 4 [Fig fig5]–6). One of the advantages of LDS is that it can assess the vibration of the target object without an accelerometer [13–15]. The attachment of the accelerometer increases the total mass of the simulated tooth (Groups A and B). The increased mechanical parameter values observed in Group C may be caused by the decrease in mass of the simulated tooth. A recent study [16] has shown that more accurate mechanical parameters were obtained when using LDS with NEVD compared to using accelerometer with NEVD in the experimental bone destruction models. This report suggested that the actual total mass of the simulated tooth due to the absence of accelerometer could contribute to more accurate mechanical parameters. Therefore, the present results about NEVD with LDS also may obtain more accurate mechanical parameters than those in NEVD with accelerometer. Moreover, using LDS with NEVD could also provide better clinical operability by eliminating the attachment of an accelerometer that is connected to the FFT analyzer by the cord.

## 5. Conclusions

Within the limitations of this* in vitro* study, the following conclusions can be drawn for the evaluation of the different periodontal tissue conditions using NEVD with LDS. The use of LDS with NEVD successfully detected the differences between various conditions of the simulated periodontal ligament and alveolar bone in the experimental tooth models. In addition, more distinguishable and accurate mechanical parameters were obtained using LDS than those with conventional accelerometer. Therefore, the application of LDS may be more suitable in combination with the NEVD for the evaluation of overall periodontal tissue conditions.

## Figures and Tables

**Table 1:** Resonant frequency (kHz).

Liquid volume	Simulated bone quality	Group A	Group B	Group C
		Median (minimum/maximum)		
3.0 mL	Polyurethane	1.39 (1.38/1.41)^a,A,I^	1.38 (1.36/1.41)^a,A,I^	2.08 (2.05/2.13)^a,A,II^
	Polyurethane foam	1.13 (1.07/1.18)^b,A,I^	1.15 (1.11/1.16)^b,A,I^	1.70 (1.68/1.74)^b,A,II^

4.0 mL	Polyurethane	0.97 (0.94/1.07)^a,B,I^	0.99 (0.97/1.04)^a,B,I^	1.37 (1.30/1.46)^a,B,II^
	Polyurethane foam	0.84 (0.81/0.89)^b,B,I^	0.78 (0.74/0.84)^b,B,I^	1.06 (1.01/1.10)^b,B,II^

5.0 mL	Polyurethane	0.69 (0.68/0.71)^a,C,I^	0.68 (0.65/0.70)^a,C,I^	1.01 (0.98/1.04)^a,C,II^
	Polyurethane foam	0.64 (0.61/0.66)^b,C,I^	0.61 (0.60/0.63)^b,C,I^	0.90 (0.89/0.96)^b,C,II^

Identical lowercase letters between polyurethane and polyurethane foam values at the same liquid volume indicate that the values within groups are not significantly different (*P* > 0.05).

Identical uppercase letters among liquid volumes with the same bone quality indicate that the values within groups are not significantly different (*P* > 0.05).

Identical Roman numerals among groups with the same liquid volume and bone quality indicate that the values are not significantly different (*P* > 0.05).

**Table 2 tab2:** Elastic modulus (×10^5^ Pa).

Liquid volume	Simulated bone quality	Group A	Group B	Group C
		Median (minimum/maximum)		
3.0 mL	Polyurethane	1.21 (1.19/1.25)^a,A,I^	1.19 (1.15/1.22)^a,A,I^	2.02 (1.97/2.12)^a,A,II^
	Polyurethane foam	0.79 (0.72/0.87)^b,A,I^	0.83 (0.77/0.85)^b,A,I^	1.36 (1.32/1.43)^b,A,II^

4.0 mL	Polyurethane	0.59 (0.56/0.72)^a,B,I^	0.61 (0.59/0.68)^a,B,I^	0.88 (0.79/1.00)^a,B,II^
	Polyurethane foam	0.44 (0.41/0.49)^b,B,I,II^	0.38 (0.35/0.44)^b,B,I^	0.53 (0.48/0.57)^b,B,II^

5.0 mL	Polyurethane	0.30 (0.29/0.31)^a,C,I^	0.29 (0.27/0.31)^a,C,I^	0.48 (0.45/0.51)^a,C,II^
	Polyurethane foam	0.24 (0.22/0.27)^b,C,I^	0.24 (0.21/0.25)^b,C,I^	0.38 (0.37/0.44)^b,C,II^

Identical lowercase letters between polyurethane and polyurethane foam values at the same liquid volume indicate that the values within groups are not significantly different (*P* > 0.05).

Identical uppercase letters among liquid volumes with the same bone quality indicate that the values within groups are not significantly different (*P* > 0.05).

Identical Roman numerals among groups with the same liquid volume and bone quality indicate that the values are not significantly different (*P* > 0.05).

**Table 3:** Coefficient of viscosity (Pa·s).

Liquid volume	Simulated bone quality	Group A	Group B	Group C
		Median (minimum/maximum)		
3.0 mL	Polyurethane	4.94 (4.75/5.31)^a,A,I^	5.06 (4.80/5.31)^a,A,I^	7.38 (7.13/8.04)^a,A,II^
	Polyurethane foam	4.12 (3.93/4.19)^b,A,I^	4.00 (3.87/4.18)^b,A,I^	4.44 (4.43/4.86)^b,A,II^

4.0 mL	Polyurethane	4.13 (3.87/4.45)^a,B,I^	3.87 (3.61/4.28)^a,B,I^	6.36 (6.36/6.82)^a,B,II^
	Polyurethane foam	3.56 (3.53/3.88)^b,B,I^	3.54 (3.30/3.67)^b,B,I^	4.11 (4.02/4.20)^b,B,II^

5.0 mL	Polyurethane	3.25 (3.12/3.43)^a,C,I^	3.18 (3.06/3.37)^a,C,I^	3.93 (3.83/4.02)^a,C,II^
	Polyurethane foam	2.87 (2.69/3.06)^b,C,I^	2.87 (2.75/3.00)^b,C,I^	2.90 (2.80/2.99)^b,C,I^

Identical lowercase letters between polyurethane and polyurethane foam values at the same liquid volume indicate that the values within groups are not significantly different (*P* > 0.05).

Identical uppercase letters among liquid volumes with the same bone quality indicate that the values within groups are not significantly different (*P* > 0.05).

Identical Roman numerals among groups with the same liquid volume and bone quality indicate that the values are not significantly different (*P* > 0.05).
